# Restructuring BOD : COD Ratio of Dairy Milk Industrial Wastewaters in BOD Analysis by Formulating a Specific Microbial Seed

**DOI:** 10.1100/2012/105712

**Published:** 2012-08-22

**Authors:** Purnima Dhall, T. O. Siddiqi, Altaf Ahmad, Rita Kumar, Anil Kumar

**Affiliations:** ^1^Environmental Biotechnology Division, Institute of Genomics and Integrative Biology, Delhi 110007, India; ^2^Botany Department, Jamia Hamdard University, Hamdard Nagar, New Delhi 110062, India; ^3^Patent Division, National Institute of Immunology, Aruna Asaf Ali Marg, New Delhi 110067, India

## Abstract

BOD (Biochemical oxygen demand) is the pollution index of any water sample. One of the main factors influencing the estimation of BOD is the nature of microorganisms used as seeding material. In order to meet the variation in wastewater characteristics, one has to be specific in choosing the biological component that is the seeding material. The present study deals with the estimation of BOD of dairy wastewater using a specific microbial consortium and compares of the results with seeding material (BODSEED). Bacterial strains were isolated from 5 different sources and were screened by the conventional BOD method. The selected microbial seed comprises of *Enterobacter* sp., *Pseudomonas* sp. BOD : COD (Chemical oxygen demand) ratio using the formulated seed comes in the range of 0.7-0.8 whereas that using BODSEED comes in the ratio of 0.5-0.6. The ultimate BOD (UBOD) was also performed by exceeding the 3-day dilution BOD test. After 90 days, it has been observed that the ratio of BOD : COD increased in case of selected consortium 7 up to 0.91 in comparison to 0.74 by BODSEED. The results were analyzed statistically by *t*-test and it was observed that selected consortium was more significant than the BODSEED.

## 1. Introduction

Dairy industry is found all over the world, but their manufacturing process varies tremendously [1]. This sector generates huge volume of wastewater and its pollution is primarily organic [2, 3]. It generates about 0.2–10 liters of effluent per liter of milk processed [4]. In general, liquid waste in dairy industry presents the following characteristics high organic content, high oils and fats content, high level of nitrogen and phosphorous, dissolved sugar, nutrients, and so forth [5].

Due to the presence of high organic load, dairy effluents degrade rapidly and deplete the DO (dissolve oxygen) level of the receiving streams and become the propagation place for mosquitoes and flies carrying malaria and other perilous disease like dengue fever, yellow fever, and chicken guinea [6, 7]. Nutrients present in dairy effluent like nitrogen and so forth lead to eutrophication of receiving waters, and detergents affect the aquatic life [3, 8]. Presence of nitrogen in dairy effluent is another major problem that once converted may contaminate ground water with nitrate [9]. Milk with 3.7% fat contains about 295 mg/L of nonprotein nitrogen [10–12]. Raw milk contains 3–8 mg/L of ammonia nitrogen [12–14] and presence of 50 mg/L of nitrogen in wastewater stream is due to 1% loss of milk [12]. Nitrogen either in the form of nitrate, nitrite, or ammonia can be health hazard. Presence of nitrate can cause methemoglobinemia if converted to nitrite [3, 15, 16] and might contaminate groundwater [17]. Nitrite can also cause intestinal cancer [18]. 

Strict guidelines have been established by government agencies to prevent water contamination [3, 8]. It is necessary to monitor the wastewater properly before discharge.

Among the wastewater parameters, BOD is widely used as a primary indicator to gauge water pollution. BOD provides information about the amount of biodegradable substance present in wastewater. As this is a bioassay test, the results depend not only on the kinetics exerted during the incubation period, but also on the microorganisms used; thus, the test exhibits poor repeatability. Some of the industrial wastewaters have sufficient microbial population to perform the BOD_5_ test without providing an acclimated microbial seed. In comparison, there are other types of wastes, namely, untreated industrial wastes, disinfected wastes, and wastes that have been treated to a high temperature, that contain negligible bacterial population to perform the test. Thus, these samples need to be seeded with a population of microorganisms to exert an oxygen demand. Seeding is a process in which the microorganisms that oxidize the organic matter present in a wastewater are added to the BOD bottle. Pierce etal. [19] have stated that measurement of very low BOD concentrations is also facilitated by the use of standard seeding material. It has been confirmed that seeding in the BOD test, and in particular the source of seeding material, is a possible major source of error [20]. In the conventional BOD test, the seed cultures are procured from sewage/domestic wastes at different times. It is well documented in the literature that sewage/activated sludge is used by most of the workers for the biodegradation of individual samples [21]. However, variations have been observed in BOD values when bacterial populations for seeding were procured either from different sources or from the same source at different time intervals. Possibly this is due to variations in the number and types of microbial population in sewage samples and/or changes in composition of bacterial population during different time intervals. For all sources of seed, the possibility exists that some wastes will not be able to degrade by the microorganisms. 

The BOD_5_ values of dairy wastewaters are often misleading since the normal seeding materials used for BOD_5_ estimation are nonspecific bacteria that cannot biodegrade some of the nitrogenous compounds present in the effluent. Pepper et al. [22] stated that the bioavailability of compounds in a given system is a very important factor determining the biodegradability of the system. Some of these compounds are refractory to biodegradation because of high molecular weight coupled with lesser bioavailability. The BOD analysis of dairy wastewater is problematic for many reasons; these include the heterogeneity of the samples at different times and nonspecific microorganism's present in general seeding material. 

The aforementioned problems can be overcome by formulating a uniform microbial seed comprising selected bacterial isolates thatare acclimatized to the dairy industrial wastewater. Further, these bacterial isolates must be specific for the biodegradation of the organic compounds present in dairy effluent. Reproducible and reliable results may be obtained if a specifically designed formulated microbial consortium comprising selected bacterial strains is used as seed for the BOD_5_ analysis.

The objective of this study is to isolate autochthonous bacteria from the industrial premises in order to develop a microbial consortium specifically formulated for use as seeding material for the BOD_5_ analysis of dairy industrial wastewater which will incorporate the utilization of nitrogen present in the dairy effluent. Screening is done by conventional method and compared with already available seeding material. Statistical *t*-test is used to check the significance of the developed consortium. Identification of the strains was done by IMTECH Chandigarh. Specific consortium was formulated so that the treatment can be effective and wastewater can be discharged after proper treatment.

## 2. Materials and Methods

### 2.1. Chemicals

D-glucose and D-glutamic acid were obtained from Sigma, Germany. Charged nylon membrane (SIGMA) with a pore size of 0.45 *μ*m was used throughout the investigation. All chemicals used to prepare the growth medium were procured from Hi-Media, India.

### 2.2. Sample Collection

5 samples specially sludge samples and sample from equalization tank were collected from different sources. Different sources are Equalization tank (MET), aeration tank (sludge) (MAT), sludge (MS), inlet sludge (MIS), clarifier (MC). Samples were stored at 4°C and analyzed within 24 hr.

### 2.3. Isolation of Bacterial Isolates from the Source Habitat 

#### 2.3.1. Soil Extract Preparation and Preparation of Enrichment Media

Different extracts were prepared for different media mentioned in (Table 1). Extracts were autoclaved at 15 psi for 1 hr. Supernatant was collected leaving pellet in a sterilized glass bottle. This supernatant was then used as Soil Extract.

The collected samples were enriched for the autochthonous bacterial population present theirin, by adding 5 gm of the sample in medium containing milk, tryptone, lactose, and soil extract. Extract used is different for different samples. This suspension was incubated at 37°C for 2 days under gentle shaking (150 rpm). Different media were designed (Table 2(a)) to isolate the bacterial strains from the above mentioned enrichment samples using serial dilution method. Serial dilutions for this purpose were prepared from 10^−1^ to 10^−12^ in 0.85% saline. One hundred microlitre of each dilution were plated on different media as listed in (Table 2(b)) and plates were incubated at 37°C for 16 hrs. Pure bacterial strains were obtained after successive transfer of individual colony in TYG (tryptone yeast and glucose) plates and incubated for 16 hrs at 37°C temperature. The contents of the medium were sterilized by autoclaving at 121°C for 15–20 minutes.

#### 2.3.2. Conventional COD and BOD 5-Day Test

The chemical oxygen demand (COD) and Biochemical oxygen demand 5-day (BOD) tests of effluent sample were carried out according to the method described in standard methods for examination of water and wastewaters. COD: A sample is refluxed in strongly acid solution with a known excess of potassium dichromate. After digestion, the remaining unreduced dichromate is titrated with ferrous ammonium sulfate to determine the amount of potassium dichromate consumed and the oxidizable matter is calculated in terms of oxygen equivalent. BOD: The method consists of filling with sample. To overflowing, an airtight bottle of the specified size and incubating it at the specified temperature for 5 days. Dissolved oxygen is measured initially and after incubation, and the BOD is computed from the difference between initial and final DO [23, 24]. 

#### 2.3.3. Screening of Individual Bacterial Isolates for BOD Removal Efficiency

The bacterial strains selected as stated above were individually inoculated in 25 mL of TYG (tryptone yeast glucose). All the cultures were incubated at 37°C for 16–20 hrs at 150 rpm. Cells were harvested by centrifugation at 7000 rpm for 15–20 min. The pellet thus obtained was washed twice with 50 mM phosphate buffer, pH 6.8. The cell pellet of individual bacterial isolates thus obtained was resuspended in 2 mL of same buffer and used as seeding material for the BOD analysis of dairy wastewater. 

#### 2.3.4. BOD Analysis of Dairy Wastewater Using Different Formulated Microbial Consortium

The inoculums was prepared by inoculating one loopful of all the individual bacterial isolates separately in 25 mL sterilized nutrient broth. The inoculated broths were incubated in an orbital shaker at 35°C for 16–24 hours so as to obtain actively growing mother cultures. After achieving the desired growth (1.2 optical density), the cultures were centrifuged at 7000 rpm for 15 min at 4°C. The cell pellet of individual bacterial isolates thus obtained was resuspended in 2 mL of same buffer and mixed at the time of performing BOD analysis of dairy wastewater. Twenty consortia were designed from 13 selected isolates. Out of 20 microbial consortia prepared for BOD analysis (APHA 1998). Three microbial consortia were selected, which exhibited the best values for dairy wastewater.

### 2.4. Stability and Reproducibility Studies

Formulated microbial consortium was tested for reproducibility by testing the wastewater collected at different periods of time with the best identified consortium.

### 2.5. Ultimate BOD Analysis of Dairy Wastewater using Selected Consortium and Its Comparison with Commercially Available BODSEED

The ultimate BOD test is an extension of the 5-d dilution test. Formulated microbial consortium was compared with BODSEED by performing ultimate BOD for 90 days [20].

### 2.6. Statistical Analysis

To statistically analyze the data *t*-test was used. Test was used to analyze the significance difference between consortia and conventional BOD values.

### 2.7. Identification of the Selected Microbial Consortium

The selected organisms of the consortium were identified by Microbial Type Culture Collection at IMTECH, Chandigarh, India.

## 3. Results and Discussion

Alexandra in 1994 defined biodegradation as the biologically catalyzed reduction in complexity of chemical compound [25]. Microorganisms either takes organic pollutant as a sole source of carbon or else degrade organic compound in the presence of growth substrate, that is, use primary carbon as a source of energy. During the decomposition process the DO in the receiving water may be utilized at a greater rate than it can be replenished, causing oxygen depletion, which has severe consequences for the stream biota. Prevention of all these adverse consequences can be done by adopting efficient water pollution management strategies. Quantitative measurement of pollutants is necessary before water pollution can be effectively managed. Microorganisms are used in the monitoring procedures from last to many years. They are the eco-friendly degraders of the organic matter. 

Industrial wastes are probably the greatest single water pollution problem as they contain large fraction of organic matter which acts as substrate for microorganisms when released in to water course. 

Dairy wastewater is of great concern due to the presence of high nitrogenous load. The conventional estimation of biological oxygen demand estimates the load in 5 days, and as per rule, we will get the results in the form of carbonaceous demand and nitrogenous demand which requires 90 days for the measurement. So in order to avoid that, the consortia was designed which will contain the bacteria which is able to give you the nitrogenous demand in 5 days.

### 3.1. Isolation of Autochthonous Bacteria

After isolation of 25 bacterial isolates were chosen randomly from all 45 bacterial isolate on the basis of their growth rate. Selected individual bacterial isolate were then used as seeding material for estimating BOD of inlet dairy industrial wastewater. The BOD in all cases was assessed and the results are presented (Table 3). 

### 3.2. Screening of Single Isolates and Consortia

**Figure 1:**
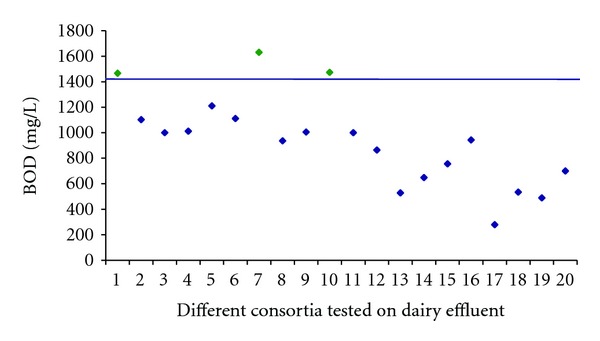
Comparison of BOD values (mg/L) of dairy wastewater sample using different formulated bacterial consortia. BOD limit for selection of consortia depicted blue color (1405 mg/L) as calculated by using BODSEED. Selected consortia above the BOD limit are shown in green.

The pollutional strength of wastewater can be estimated by measuring oxygen demand. Primary parameters for monitoring wastewater quality are COD and BOD. COD gives the total load either in the form of organic or inorganic. It cannot differentiate between the two loads, or we can say COD tells us the total pollutional load of wastewater. The BOD test has been widely measured the organic load of wastewater in terms of carbonaceous matter. So we can say it can give a far more reliable estimation of the possible oxygen demand that a waste will have on a river than a COD test. So we can define BOD as a measure of oxygen required for the biochemical oxidation of the organic matter. Although the BOD test is not specific to any pollutant, yet it continues to be one of the important general indicators of the potential of a substance for environmental pollution of surface waters. For screening the single isolates and consortia BOD was performed. Those individual bacterial isolates, which exhibited BOD values higher to or comparable to BODSEED, were chosen. Out of the above screened isolates, 14 bacterial isolates (2, 5, 6, 7, 9, 10, 11, 12, 13, 14, 19, 23, 24, and 25) were selected for the formulation of different microbial consortia. In the subsequent experiment, seeding was carried out at 0.1% as in the case being done with BODSEED. The results of the BOD analysis performed using the microbial seeds (20 consortia) are illustrated in figure (Figure 1).

### 3.3. Screening of Selected Consortia

**Figure 2:**
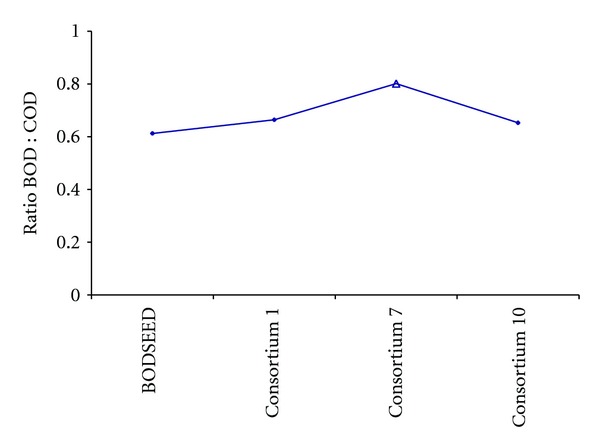
Comparison of BOD : COD ratios of dairy industrial wastewater sample (*n* = 3) using 3 selected bacterial consortia. Data shown with symbol Δ represent the best performing consortia.

On the basis of the results obtained in the above experiment, further selections were carried out according to the ability of the screened consortia to biodegrade the constituents of dairy industrial wastewater. The selected bacterial consortia were again tested for the BOD analysis of a fresh lot of dairy industrial effluent. Out of 20 bacterial consortia selected for the BOD analysis, 3 consortia, which exhibited the best values for dairy effluent, were selected (Figure 2). 

BOD : COD ratios exhibited by the above 3 consortia showed that the ratio can be increased with the help of selected and screened bacteria.

### 3.4. Stability and Reproducibility Studies

**Figure 3:**
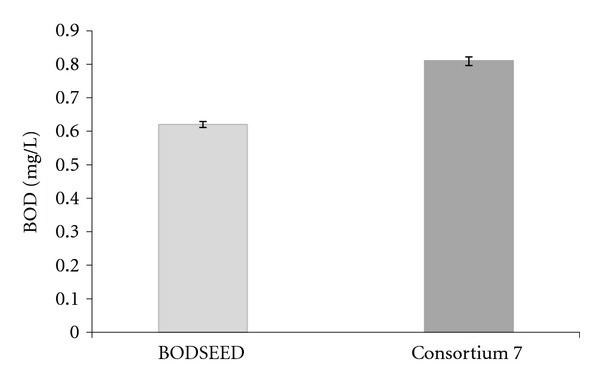
BOD analysis of dairy industrial wastewater sample using the selected bacterial consortium 7 (*n* = 5).

 It was evident from the results that consortium 7 was performing the best in all the experiments conducted during the course of the study. The BOD : COD ratios increase remarkably to 0.75–0.8 as against 0.58–0.62 obtained with the conventional seeding material. Therefore, this consortium was selected for use as seeding material, specifically for BOD analysis of dairy wastewater sampled at various time intervals over a six-month period. The results of this study are presented in (Figure 3). After performing the “*t*-test” it was observed that the consortium 7 is more significant than BODSEED used for the BOD analysis.

### 3.5. Ultimate BOD Performed by Selected Consortia

As mentioned in standards methods APHA 1998 biochemical oxygen demand estimation is divided into two groups carbonaceous oxygen demand requires 3–5 days for estimation and ultimate oxygen demand (carbonaceous + nitrogenous demand) requires 90 days for estimation and known as ultimate BOD. 

Ultimate BOD was performed using consortium 7 and results were compared with BODSEED. The results revealed that the consortium will able to give 2005 mg/L of BOD after 90 days of incubation and BOD reaches to 1635 mg/L with BODSEED (Table 4). 

**Figure 4:**
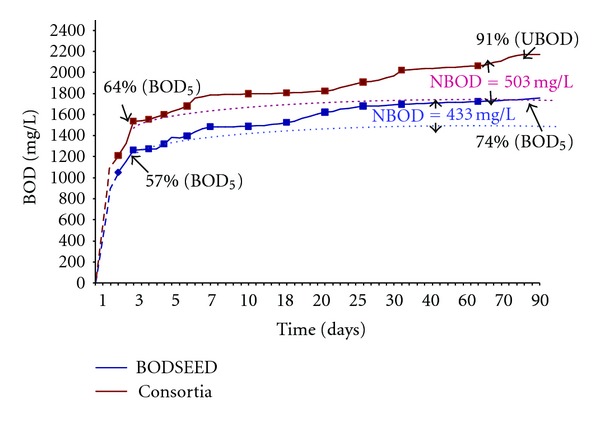
Comparison of ultimate BOD of dairy industrial wastewater sample using 3 selected bacterial consortia and conventional seeding material (BODSEED).

While comparing the ratios in case of consortia and BODSEED the BOD : COD ratio increased to 0.91 as against 0.74 obtained with the conventional seeding material (Figure 4). 

### 3.6. Statistical Analysis

It was found that both the techniques are significantly different at *P* < 0.001 (*t* = 14.37). Percentage degradation increase in consortia (0.91) while in BODSEED its degradation was 0.74 only. So, it can be concluded on the basis of percentage observed that the selected consortium was much significant than the BODSEED.

### 3.7. Identification of Selected Microbial Consortium

The bacterial strains comprised in this consortium were identified as *Enterobacter *sp. and* Pseudomonas* sp. which are deposited at international depository at IMTECH, sector 39A, Chandigarh, India.

## 4. Conclusions

BOD : COD ratio determines the biodegradability of waste water. From the above studies, it is clear that specific bacteria can be identified for degrading particular compounds present in wastewater. Moreover, the ratio of BOD : COD showed considerable increase to 0.91 as against 0.74 obtained with the conventional seeding material after 90 days of incubation at 27°C, thereby changing the degree of biodegradability of industrial waste water. 

## Figures and Tables

**Table 1:** Soil extract preparation.

Sample	Distill water
500 mL (MET/M1)	500 mL
500 mL (MAT/M2)	500 mL
1 litre (MS/M3)	—
1 litre (MIS/M4)	—
1 litre (MC/M5)	—

**Table:** (a)

Serial number	MET (M1)	MAT (M2)	MS (M3)	MIS (M4)	MC (M5)
1	M1A	M2A	M3A	M4A	M5A
2	M1B	M2B	M3B	M4B	M5B
3	M1C	M2C	M3C	M4C	M5C
4	M1D	M2D	M3D	M4D	M6D

**Table:** (b)

Medium laboratory	Medium composition
M1A	75% Soil extract + 25% Milk + Tryptone + Lactose
M1B	Soil extract + 2% Agar
M1C	75% Soil extract + 25% Milk + 2% Agar
M1D	Tryptone + Yeast extract + Glucose + Dipotassium hydrogen phosphate + 2% Agar

Note: Soil extract is replaced depending upon the sample used.

**Table 3:** Comparison of BOD values (mg/L) of dairy industrial wastewater sample using individual bacterial isolates (as seeding material) and using GGA as a reference standard.

Serial number	Seeding Source (Laboratory names of Individual bacterial isolates)	BOD mg/L
1	BODSEED	1999
2	Isolate 1	100
3	Isolate 2	893
4	Isolate 3	260
5	Isolate 4	580
6	Isolate 5	1526
7	Isolate 6	1886
8	Isolate 7	1273
9	Isolate 8	426
10	Isolate 9	1000
11	Isolate 10	1530
12	Isolate 11	1875
13	Isolate 12	1840
14	Isolate 13	2185
15	Isolate 14	1605
16	Isolate 15	1230
17	Isolate 16	1245
18	Isolate 17	1175
19	Isolate 18	235
20	Isolate 19	560
21	Isolate 20	104
22	Isolate 21	369
23	Isolate 22	489
24	Isolate 23	1287
25	Isolate 24	1111
26	Isolate 25	1210

**Table 4:** Comparison of ultimate BOD values (mg/L) of dairy industrial wastewater sample using consortium 7 (as seeding material) and BODSEED (as conventional seeding material).

			COD = 2195 mg/L	
Time (in days)	BOD mg/L		Ratio BOD/COD	
			Ratio BOD mg/L/COD mg/L	
	With BODSEED	With Consortia	BODSEED/COD	Consortia/COD
1	1049	1210	0.48	0.55
3	1262	1412	0.57	0.64
5	1276	1435	0.58	0.65
7	1322	1601	0.60	0.73
10	1345	1633	0.61	0.74
15	1399	1674	0.64	0.76
18	1425	1712	0.65	0.78
20	1463	1768	0.67	0.81
25	1498	1795	0.68	0.82
30	1515	1856	0.69	0.85
40	1549	1912	0.71	0.87
50	1578	1935	0.72	0.88
60	1610	1968	0.73	0.90
70	1629	1985	0.74	0.90
90	1635	2005	0.74	0.91
